# Endometriosis-Associated Ovarian Carcinomas: How PI3K/AKT/mTOR Pathway Affects Their Pathogenesis

**DOI:** 10.3390/biom13081253

**Published:** 2023-08-16

**Authors:** Tatiana S. Driva, Christoph Schatz, Johannes Haybaeck

**Affiliations:** 1 First Department of Pathology, Medical School, National and Kapodistrian University of Athens, 11527 Athens, Greece; 2 Institute of Pathology, Neuropathology and Molecular Pathology, Medical University of Innsbruck, 6020 Innsbruck, Austria; 3 Diagnostic & Research Center for Molecular Biomedicine, Institute of Pathology, Medical University of Graz, 8010 Graz, Austria

**Keywords:** endometrium, endometriosis, ovarian cancer, PI3K/AKT/mTOR pathway, ARID1A mutations

## Abstract

Ovarian clear cell (OCCC) and endometrioid (EnOC) carcinomas are often subsumed under the umbrella term “endometriosis-associated ovarian cancer” (EAOC), since they frequently arise from ectopic endometrium settled in the ovaries. The phosphatidylinositol 3-kinase (PI3K)/protein kinase B (AKT)/mammalian target of rapamycin (mTOR) pathway is known to be aberrantly activated both in endometriosis and EAOC; however, its role in the progression of endometriosis to ovarian cancer remains unclear. In fact, cancer-associated alterations in the mTOR pathway may be found in normal uterine epithelium, likely acting as a first step towards ovarian cancer, through the intermediary stage of endometriosis. This review aims to summarize the current knowledge regarding mTOR signaling dysregulation in the uterine endometrium, endometriosis, and EAOC while focusing on the interconnections between the PI3K/AKT/mTOR pathway and other signaling molecules that give rise to synergistic molecular mechanisms triggering ovarian cancer development in the presence of endometriosis.

## 1. Introduction

Endometriosis is a disease characterized by the ectopic implantation and development of endometrial tissue outside the uterus, concerning approximately 10% of women of reproductive age worldwide [1,2]. Despite being considered a benign condition, endometriosis may undergo malignant transformation and progress to certain ovarian cancer subtypes [3] that are believed to arise from ovarian endometrial cysts (ECs) [4]. In fact, the relative risk for ovarian cancer development in the presence of endometriosis might be up to 1.42 [5]. Endometriosis-associated ovarian cancer (EAOC) comprises mostly endometrioid or clear cell carcinoma histotypes, the presence of which is more likely to co-exist with endometriosis [6,7].

The mammalian target of the rapamycin (mTOR) pathway is a key modulator of cell proliferation, growth, apoptosis, and angiogenesis, balancing cellular resources like amino acids and growth hormones with stressors like hypoxia to control cellular behavior [8]. Due to its prominent role in cell cycle progression, derangement of mTOR signaling is implicated in a wide range of malignancies [9], including ovarian cancer [10], while it has also been proven to correlate with endometriosis [11]. Despite this evidence, the function of mTOR in the transformation process of endometriosis into ovarian cancer remains poorly understood.

In this review, we discuss the role of the mTOR pathway in the establishment and malignant progression of endometriosis, with a focus on its interplay with other molecular pathways involved in EAOC development.

## 2. Outline of mTOR Signaling and Its Function in Normal Endometrium

mTOR signaling plays a critical role in the coordination of numerous cellular properties, as it regulates cell metabolism, growth, and proliferation, while also controlling autophagy and apoptosis [12]. Specifically, the mTOR protein acts as a serine/threonine protein kinase [13] and can be part of either one of two discrete complexes: the mammalian target of rapamycin complex 1 (mTORC1) or the mammalian target of rapamycin complex 2 (mTORC2) [14]. The first one comprises mTOR, Raptor, mLST8/GL, PRAS40, and DEPTOR, while mTORC2 comprises mTOR, Rictor, mLST8/GL, PRR5, DEPTOR, and SIN1. These complexes not only differ in formation but are also characterized by a distinct functional potential.

mTORC1 function, which has been investigated the most, is known to be triggered by a multitude of stimuli coming from the environment, including nutrients, oxygen deprivation, energy supply, and growth factors [15], the latter being its most critical regulators [8]. In brief, after growth factors like insulin bind to their membrane receptors, phosphatidylinositol-3-kinase (PI3K) becomes activated and subsequently turns phosphatidylinositol-4,5-phosphate into phosphatidylinositol-3,4,5-phosphate (PIP3), a procedure hampered by tumor suppressor protein phosphatase and tensin homolog (PTEN) [16]. Next, PIP3 promotes the activation of protein kinase B (AKT), which, in turn, impedes the formation of the tuberous sclerosis complex (TSC), the major negative regulator of mTORC1, thereby permitting mTORC1 to become stimulated [17]. Importantly, mTORC1 can be inactivated by adenosine monophosphate (AMP)-activated protein kinase (AMPK), a protein that is responsive to alterations of cellular energy levels. AMPK inhibits mTORC1 signaling via activation of TSC or by Raptor suppression [15].

When stimulated, mTORC1 is able to exert its two key biological functions, namely the promotion of protein synthesis and the inhibition of autophagy. The first begins with the phosphorylation of eIF4E Binding Protein 1 (4EBP1) and p70S6 Kinase 1 (S6K1). After being phosphorylated by mTORC1, S6K1 mediates both translation initiation and elongation by activating the translation initiation factor 4B (eIF4B) and regulating eukaryotic elongation factor 2 kinase (eEF2K) activity, respectively [18]. Furthermore, mTORC1-mediated phosphorylation of 4EBP1 causes the latter to detach from the eukaryotic translation initiation factor 4E (eIF4E), thereby letting eIF4E become a component of the eIF4F complex and participate in translation initiation [19]. Secondly, mTORC1 impedes the procedure of autophagy by suppressing the factors ATG13 and ULK1/2 [20].

Unlike mTORC1, little is known about mTORC2’s properties and regulation. Most importantly, mTORC2 acts by phosphorylating AKT, while also activating multiple AGC family members, thus controlling the structure of the cytoskeleton and cell migration [21,22]. In contrast to mTORC1, the activity of mTORC2 is actually promoted by the TSC complex [17]. Moreover, mTORC2 is not susceptible to acute rapamycin administration, although it may be suppressed following long-term treatment with this agent [23].

mTOR signaling is of high importance for the maintenance of endometrial homeostasis, as it is involved in all complex procedures included in the cyclical and rhythmic remodeling of endometrial tissue, such as cell proliferation, apoptosis, autophagy, differentiation, and decidualization [24,25]. In fact, estrogen regulates DNA and protein synthesis in endometrial cells via the mTOR pathway, thereby finally controlling mitosis and cell differentiation in the endometrium [11,24]. Additionally, apoptosis of endometrial stromal cells (ESCs) before menstruation results from autophagy induction, which is mediated by mTOR signaling suppression [26,27]. In further detail, normal eutopic endometrial tissue exhibits increased levels of cleaved caspase-3 and decreased levels of phosphorylated S6K1 during the late secretory phase of the menstrual cycle, meaning that endometrial cell apoptosis is associated with autophagy caused by mTOR inhibition [26]. Moreover, nm23-mediated PI3K-Akt-mTOR pathway activation in mouse- and human-derived ESCs has been shown to promote decidualization, the process where ESCs are transformed into specialized secretory decidual cells. Nm23 is a protein with regulatory properties in cell proliferation and differentiation and is known to act as a metastasis suppressor [28,29]. Other than that, endometrial atrophy during menopause has been shown to result from PI3K/AKT/mTOR pathway downregulation, as a consequence of the estrogen withdrawal that characterizes this period [25]. Therefore, since mTOR signaling plays a vital role in endometrial function during the different stages of human female life, its derangement would be expected to have a negative effect on the integrity of endometrial tissue.

**Figure 1:**
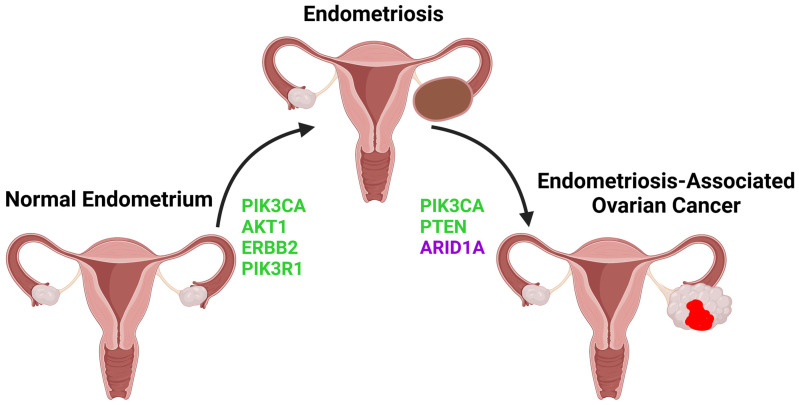
PI3K/AKT/mTOR pathway-related gene alterations involved in the pathogenesis of Endometriosis and Endometriosis-Associated Ovarian Cancer. Normal uterine epithelium harboring activating mutations in PIK3CA, AKT1, ERBB2, and PIK3R1 and is correlated with a higher tendency towards endometriosis development [30,32,33,34], while endometriotic epithelium carrying additional activating mutations in PIK3CA and inactivating mutations in PTEN and ARID1A is more likely to undergo malignant transformation [35]. Molecules depicted in green are components of the PI3K/AKT/mTOR signaling pathway. ARID1A, depicted in purple, does not actively participate in mTOR signaling, but closely interacts with it, in such a way that loss of ARID1A expression probably allows the function of the mTOR pathway, which is enhanced by the aforementioned genetic alterations, to induce carcinogenesis in ovarian endometriotic cysts. PIK3CA, Phosphatidylinositol-4,5-bisphosphate 3-kinase catalytic subunit alpha; AKT1, RAC-Alpha serine/threonine-protein kinase; ERBB2, Human epidermal growth factor receptor 2; PIK3R1, Phosphoinositide-3-kinase regulatory subunit 1; PTEN, Phosphatase, and tensin homolog; ARID1A, AT-rich interaction domain 1A.

Interestingly, normal uterine epithelial cells have been shown to harbor cancer-driver alterations in genes associated with the mTOR pathway. For instance, PIK3CA has been found to be the most frequently mutated gene in normal eutopic endometrial glands, carrying hotspot mutations directly related to carcinogenesis [30], while focal loss of PTEN expression has also been observed in the histologically normal uterine epithelium [31]. Additionally, driver mutations have been found in genes coding for upstream effectors of the PI3K/AKT/mTOR pathway, such as growth factor receptors like ERBB2, ERBB3, and FGFR2 [32]. As will be further analyzed in this review, similar genetic alterations in mTOR pathway components occur in both eutopic and ectopic endometrium of endometriosis patients as well as in endometriosis-associated ovarian cancer. This may suggest that spontaneous and repeated mTOR pathway-associated mutations arising throughout normal endometrial glands may act as precursor events that, upon the emergence of additional genetic abnormalities, can lead to endometriosis-associated ovarian cancer, via the intermediary state of endometriosis (Figure 1).

## 3. How mTOR Signaling Affects Endometriosis Development

Endometriosis is a condition in which endometrial tissue grows in areas outside the uterine cavity, such as the pelvic peritoneum, ovaries, and pouch of Douglas [36]. It is a disorder that emerges upon an increase in the proliferative, invasive, and migratory potential of phenotypically normal endometrial cells, thereby displaying cancer-mimicking features, right from the onset of its development. Among various signaling pathways that control cell proliferation, PI3K/AKT/mTOR pathway is being studied as a possible component of the pathogenetic mechanism that leads to endometriosis [37]. In fact, endometriosis patients exhibit higher levels of phosphorylated AKT in both eutopic and ectopic endometrial stromal cells, when compared with healthy controls [38]. Additionally, AKT1, 4EBP1, and the mTOR activators AXL and SHC1 have been shown to be highly expressed in eutopic and ectopic endometrial tissue, respectively, all of them in both epithelial and stromal cells [39,40], while PTEN loss has been described in ectopic endometrial epithelial cells already in early studies [41].

More recently, Madanes et al. observed higher PI3K and phosphorylated AKT levels as well as lower levels of PTEN expression in both eutopic and ectopic endometrium of women with endometriosis compared to controls. Importantly, these findings concerned patients at a minimal to mild stage of endometriosis, proposing that mTOR signaling activation has a key role in the initiation of the disease, by triggering the development of ectopic lesions [42]. The results of a mouse model experiment conducted by Kim et al. were supportive of this conclusion, as they showed a rise in the establishment of endometriosis lesions in mice harboring a deletion of PTEN in PR-positive cells [43]. In the same study, subsequent treatment of the mice with the allosteric AKT inhibitor MK-2206 led to a significantly reduced number of formed lesions, thus demonstrating the importance of mTOR signaling in the pathogenesis of endometriosis. The epithelial compartment of deep infiltrating endometriosis lesions has also been shown to carry hotspot mutations in PIK3CA, a catalytic subunit of PI3K, and PTEN at significant rates and these single mutational hits are thought to be inherent features of the disease [34,44].

In addition to the intensity of mTOR signaling in endometriotic lesions, mTOR activity has been shown to remain constant in endometriotic cyst stromal cells, despite changes in estrogen and progesterone levels that can be observed during the menstrual cycle or in cases of exogenous hormone administration [24,26]. Moreover, endometriosis is frequently accompanied by resistance to progesterone, a condition that may also be associated with PI3K/AKT/mTOR pathway activation, as the latter seems to modulate the response of ectopic endometrium to progesterone [45,46]. In fact, the inhibition of AKT elevates progesterone expression in endometriotic stromal cells [43], while PTEN suppression by the microRNA miR-92a leads to progesterone-resistant endometriosis, according to the study of Li et al. [47].

Aside from miR-92a, two other microRNAs appear to be involved in mTOR pathway regulation in endometriosis. Zhao et al. explored the role of miR-194-5p, a microRNA that represses mTOR signaling by downregulating STAT1 expression, which was found to be under-expressed ectopic endometrial epithelial cells in mouse models of the disease [48]. Furthermore, Zhou et al. reported that miR-106a-5p inhibits ectopic endometrial stromal cell proliferation, migration, and invasion, probably by suppressing the PI3K/AKT/mTOR signaling cascade after inactivating the protein FOXC1 [49].

On account of all these findings, PI3K/AKT/mTOR pathway has emerged as an appealing therapeutic target in endometriosis, with scientists attempting to assess the effects of its blockade on the progression of the disease. In fact, dienogest, which is a progestin drug approved for endometriosis treatment, works by downregulating mTOR signaling, and specifically by repressing the activity of ERK1/2 and AKT, thereby promoting apoptosis and autophagy in human endometriotic cyst stromal cells [50]. In support of this concept, Leconte et al. noted that temsirolimus-induced mTOR inhibition resulted in a statistically significant decrease in deep infiltrating endometriotic cell proliferation in a dose-dependent manner, both in vitro—only stromal cell lines were tested—and in vivo [51]. In addition, Ren et al. demonstrated that the well-known mTOR inhibitor rapamycin may be able to inhibit the angiogenesis occurring in endometriosis, as they observed a marked reduction in VEGF levels and the density of microvessels in ectopic lesions of mice with peritoneal endometriosis following 2-week treatment with rapamycin [52]. In the same spirit, Cao et al. indicated the ability of the vasodepressor ginsenoside Rg3 to slow down the growth rate of ectopic endometrium via inhibition of mTOR signaling activation by VEGF [53].

## 4. The role of mTOR Signaling in Endometriosis-Associated Ovarian Carcinomas (EAOCs)

### 4.1. PI3K/AKT/mTOR Pathway Alterations in EAOC

Ovarian endometriotic lesions entail a risk of malignant transformation and are associated with an increased likelihood of progressing to epithelial ovarian carcinoma (EOC), especially to clear cell or endometrioid histotypes [54]. Since mTOR pathway alterations seem to occur as an early event in the onset of endometriosis, it would not be surprising that continuous misfunction of this proliferation-inducing signaling can increase the malignant potential of endometriotic cells. In fact, as already mentioned, whole exome sequencing studies have revealed that many of the PIK3CA mutations found in the normal uterine and endometriotic epithelium are non-silent and often coincide with cancerogenic mutations [30] (Figure 1), while at the same time, the most frequent gene alterations in ovarian clear cell carcinoma (OCCC) affect the KRAS/PI3K pathway (82%) [55]. It is thus likely that these PIK3CA mutations are functionally significant and might act as a first step towards ovarian cancer through the intermediary stage of endometriosis. Consistent with this theory, Anglesio et al. demonstrated that in endometriosis-associated ovarian carcinoma (EAOC) cases carrying an activating PIK3CA mutation, the same mutation, without differences in allelic frequency, constantly appeared in both cancer and endometriosis specimens, the latter having either typical or atypical histology [56]. In the same spirit, no statistically significant difference in PTEN loss has been noted between case-matched endometriosis and OCCC samples [57]. However, as mentioned in the previous chapter, PTEN is under-expressed in most endometriosis samples, regardless of the OCCC co-occurrence. This fact suggests that whereas PTEN inactivating mutation is not sufficient to induce malignant transformation of endometriotic cells on its own, it opens the way for subsequent genetic events that may lead to carcinogenesis.

Broadway et al. proved a clearer mTOR signaling upregulation in EAOC. Specifically, they observed a significant rise of mTOR levels in endometriosis and endometrioid ovarian carcinoma (EnOC) patient samples, compared to non-affected controls and other ovarian carcinoma histotypes, respectively [7]. They also suggested the use of the mTOR-inhibitor DEPTOR as a prognostic marker for overall survival linked to ovarian cancer, since they demonstrated that DEPTOR upregulation positively correlates with better prognosis of these patients. PIK3CA activating mutations have also been significantly associated with favorable progression-free survival of EnOC patients [58]. In fact, EnOCs harbor very often PI3K pathway-associated mutations, including those affecting PIK3CA, PTEN, and PIK3R1 genes [59,60]. In particular, Pierson et al. demonstrated that more than 30% of EnOCs carry activating PIK3CA mutations, after analyzing the genetic data from a 26-EnOC cohort, as well as from 96-EnOC cases curated in the Genomics Evidence Neoplasia Information Exchange (GENIE) database [61]. Importantly, the aforementioned genetic mutations are significantly less frequent in high-grade serous ovarian carcinoma (HGSOC) and more prominent in endometrioid endometrial carcinoma compared to pure EnOC, proving the genetic heterogeneity of the latter [61].

### 4.2. The Role of ARID1A Gene Expression in EAOC

The participation of mTOR signaling in EAOC development is mostly supported by the presence of a negative correlation between PI3K/AKT/mTOR pathway activation and ARID1A gene expression. ARID1A is a tumor suppressor gene, encoding for BAF250a, a subunit of the SWI/SNF chromatin-remodeling complex, which alters the accessibility of chromatin to different nuclear factors, thereby preventing genomic instability [62,63,64]. ARID1A is mutated in a wide range of cancers, especially in those arising from ectopic or eutopic endometrium, including EAOC [62,65]. Downregulation of ARID1A has been reported already at the level of endometriosis [66,67], and in one study, this was attributed to hypermethylation of the gene promoter [68]. In fact, ARID1A under-expression is thought to induce malignant transformation of endometriotic lesions in a stepwise manner, as a gradual loss of ARID1A expression has been observed from benign (20%) to atypical endometriosis (40%) and to OCCC adjacent to endometriosis (58%), the latter also compared with papillary serous carcinoma, where the expression of wild-type ARID1A was conserved [69]. In the same vein, in a series of ARID1A-deficient EAOC samples, all of the case-matched atypical endometriosis and 86% of the case-matched non-atypical endometriosis samples exhibited loss of ARID1A immunoreactivity [70].

In contrast to this theory, Worley et al. noted no significant difference in ARID1A loss between OCCC and co-occurring endometriosis. Interestingly, they observed ARID1A under-expression in ectopic endometrial tissue, regardless of the proximity of the latter to OCCC [71]. Based on their results, the loss of ARID1A acts as an initial trigger for the malignant transformation of endometriosis tissue and arises independently of the “atypical endometriosis” phenotype. The idea that ARID1A mutation is an early event in the progression of endometriosis toward cancer has also been suggested by Wiegand et al., who have demonstrated that loss of BAF250 expression is strongly related to EAOCs [72]. Specifically, they observed ARID1A mutations in high percentages of OCCCs and EnOCs, in contrast to HGSOCs, where wild-type ARID1A expression was retained. Similarly, negative immunohistochemical expression of ARID1A has been noted in 66% of either OCCCs or EnOCs developing on endometriotic cysts, as well as in the endometriotic epithelium proximal to the tumor [73]. On the other hand, Yachida et al. reported that 16% of OCCCs and all benign endometriosis samples carrying ARID1A loss-of-function mutations preserved immunoreactivity for ARID1A [74]. These data are consistent with the “two-hit” hypothesis, meaning that both alleles of the ARID1A gene must be inactivated in order to cause a phenotypic change [74,75].

### 4.3. Synergistic Crosstalk between ARID1A and PI3K/AKT/mTOR Pathway in EAOC

Even though the loss of ARID1A expression is a major genetic event in EAOC, it probably does not exert its oncogenic effects individually but seems to be involved in crosstalk with PI3K/AKT/mTOR pathway in order to promote carcinogenesis at the ectopic endometrium. This theory is supported by an increasing body of evidence, including the study of Huang et al., where the loss of ARID1A and PTEN expression, as well as PIK3CA activation, were detected in 52%, 12%, and 34% of 68 OCCC samples, respectively, and a significant correlation between ARID1A loss and PI3K/AKT pathway activation was demonstrated [76]. Wiegand et al. showed that approximately 60% of OCCCs and EOCs carrying ARID1A inactivating mutations harbored synchronous PIK3CA activating mutations, the latter mostly concerning the kinase domain or other than the helical domain of the molecule. They also explored PI3K/AKT/mTOR pathway activation by analyzing AKT phosphorylation levels in correlation with different genetic profiles of the cancer samples. Multivariate logistic regression showed that the amount of pAKT-Thr308 was significantly elevated in carcinomas lacking BAF250a (ARID1A) immunohistochemical expression. Interestingly, tumors with BAF250a loss exhibited increased p-AKT levels, independently of alterations in PIK3CA or PTEN. However, no correlation was noted between p-AKT status and ARID1A mutations. This could be explained by the fact that BAF250a immunoreactivity may result from heterozygous mutations or epigenetic alterations of the ARID1A gene. The team further investigated the correlation between ARID1A loss and AKT phosphorylation, by examining p-AKT levels in OCCC cell lines after ARID1A knockdown. Again, they could not demonstrate a clear rise in p-AKT or its downstream effectors, suggesting that models based on cell lines may be inaccurate for reflecting the alterations in signaling that happen in real tumors, where pAKT levels may be modulated by other factors, including tumor microenvironment and coexisting gene-mutations [77].

Synchronous alterations in mTOR signaling and ARID1A expression are highly frequent in EAOCs and their precursor endometriosis lesions, reinforcing the idea that both genetic events promote EAOC development in a synergistic manner. In support of this theory, Chene et al. noted that loss of BAF250a immunoreactivity was combined with higher pAKT levels in EAOC and adjacent endometriosis tissue, compared to benign endometriosis samples [78]. According to earlier data, in OCCC, ARID1A loss often co-occurs with the PIK3CA activating mutation [79] and ZNF217 amplification [76]. The latter is an oncogene that triggers PI3K/AKT signaling by inducing ErbB3 overexpression [80]. Along the same lines, in a series of 42 OCCC samples, somatic mutations of PIK3CA were detected in 40% of the carcinomas and the majority (71%) of these were ARID1A-deficient tumors [70]. Similar findings have been mentioned for EnOC, where the loss of ARID1A and PTEN expression coexist at a statistically significant frequency [64,81].

As mentioned above, EAOCs are more common in certain Asian ethnicities, particularly in Japanese women [44]. Because of this higher prevalence, EAOC-associated genetics have been studied specifically for these populations, the findings being similar to those already described. Thus, in Japanese EAOC patients, the most frequent genetic alterations identified by DNA sequencing are activating mutations of PIK3CA and inactivating mutations of ARID1A and PTEN [82,83,84,85,86]. Additionally, in the study of Rahman et al., PIK3CA mutations were related to the favorable overall survival of Japanese OCCC patients [86]. Likewise, Itamochi et al. noted a significantly higher three-year overall survival rate for Japanese women carrying OCCCs with PI3K/AKT pathway activation, suggesting this pathway is a potential future biomarker for this type of cancer. In the studies of Er et al. and Su et al., deep sequencing for cancer-related genes was performed in EAOCs deriving from two different cohorts of Taiwanese patients [87,88]. Both studies identified ARID1A and PIK3CA as the most commonly mutated genes, carried by the majority of the tumors, followed by other genetic alterations related to the PI3K/AKT/mTOR pathway, including mTOR, AKT1, and PTEN mutations. In some cases, those genetic alterations were also identified in endometriosis coexisting with the tumor [87]. Importantly, Su et al. detected concurrent ARID1A-PIK3CA mutations in nearly 45% of samples, as well as the existence of three co-occurring gene alterations, namely ARID1A-KRAS-PIK3CA and ARID1A-CREBBP-PIK3CA [88]. In a recent Korean Gynecologic Oncology Group study, repetitive mutations in genes coding for molecules participating in the PI3K/AKT pathway like PIK3CA and PTEN were identified in OCCCs and EnOCs, indicating the therapeutic potential of PI3K-targeting treatment for these tumors [89]. In the same vein, Kim et al. discovered a high frequency of mutations in PIK3CA and ARID1A genes in a cohort of Korean OCCC patients, noting no significant difference in genetic alterations between endometriosis-associated and non-endometriosis-associated OCCCs [85]. Similarly, in a cohort of OCCCs deriving from Chinese women, the PI3K/AKT and chromatin remolding pathways were genetically altered in 83% and 71% of cases, respectively [90].

Table 1 summarizes the data about gene alterations in EAOCs that greatly affect mTOR pathway activation, while highlighting the interconnection between mTOR signaling and chromatin remodeling in EAOC development, via ARID1A-PIK3CA co-mutations.

### 4.4. The Interplay between IL-6 and the PI3K/AKT/mTOR Pathway in EAOC

**Figure 2:**
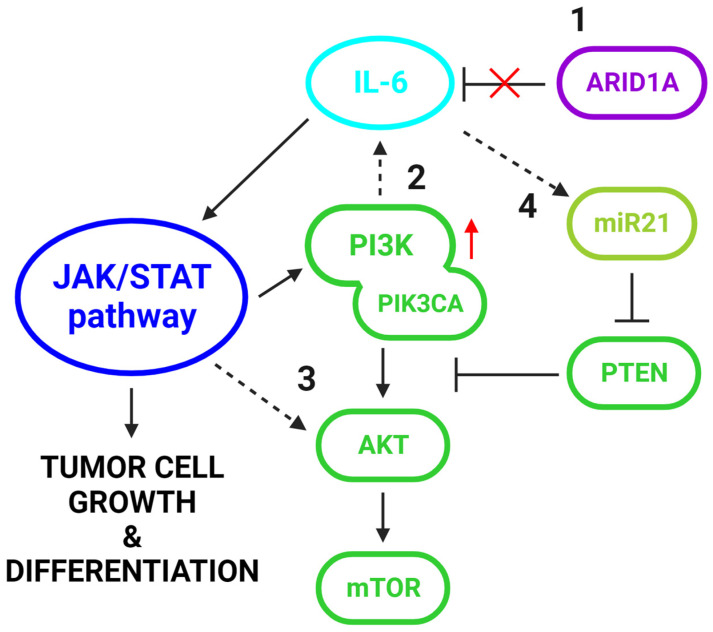
**Crosstalk between IL-6 signaling and the PI3K/AKT/mTOR pathway in EAOC.** (1) IL-6 is a physiological target of ARID1A tumor-suppressor activity, which is frequently deficient in EAOC [75]. (2) IL-6 expression is promoted by PI3K, including its catalytic subunit PIK3CA, which is commonly amplified in EAOC [101]. (3) IL-6 triggers the JAK-STAT signaling pathway, which, in turn, has been shown to interact directly with the p58a regulatory subunit of PI3K and to upregulate the expression of AKT [102,103,104]. (4) IL-6 induces the expression of miR-21, a microRNA that impedes PTEN translation and is increased in EAOC [105,106]. ARID1A, AT-rich interaction domain 1A; IL-6, Interleukin 6; PI3K, Phosphatidylinositol-3-kinase; PIK3CA, Phosphatidylinositol-4,5-bisphosphate 3-kinase catalytic subunit alpha; JAK, Janus kinase; STAT, signal transducer and activator of transcription protein; AKT, Protein kinase B; miR-21, microRNA-21; PTEN, Phosphatase, and tensin homolog.

Endometriosis-associated CCC has been considered a chronic inflammatory disease, as it exhibits high levels of proinflammatory cytokines including IL-1, IL-6, IL-8, IL-10, and TNF-α [96,97]. Among them, IL-6 seems to promote carcinogenesis by interacting with the PI3K/AKT/mTOR pathway [98]. In a mouse model experiment conducted by Chandler et al., concurrent PIK3CA and ARID1A mutations in the ovarian surface epithelium-induced rapid development of ovarian cancer, which shared histopathological features with human OCCC and was often accompanied by the presence of hemorrhagic ascites and peritoneal metastases [75]. According to this study, synchronous ARID1A loss and PIK3CA overactivation synergistically induced upregulation of IL-6, a cytokine that triggers and is triggered by the JAK/STAT pathway, thereby initiating a signaling cycle that promotes tumor cell growth and differentiation [99,100]. Specifically, they identified IL-6 as a direct target of ARID1A tumor-suppressor activity and suggested that under the absence of the negative regulation of the latter, coexisting amplification of PIK3CA promoted IL-6 overexpression, thus maintaining the JAK/STAT signaling loop [101], which in turn positively interconnects with the mTOR pathway [102,103,104]. Therefore, the synergistic action of ARID1A loss and PI3K/AKT/mTOR pathway upregulation in the malignant progression of endometriosis can be partially explained by their cooperation in activating IL-6 and thus in promoting a pro-tumorigenic inflammatory cytokine signaling (Figure 2).

Apart from being regulated by PI3K, IL-6 interacts with the PI3K/AKT pathway also by inhibiting PTEN expression. Specifically, IL-6 induces the expression of miR-21, a mi-croRNA that impedes PTEN translation [105,106] (Figure 2). Interestingly, the results of an analysis including a cohort of primary CCC tumors revealed a significant association between endometriosis-associated CCC and high miR-21 levels. The in vitro experiments following this analysis showed that the PTEN expression significantly increased following miR-21 suppression and that PTEN mRNA is a physiological target of miR-21. MiR-21 has also been demonstrated as a reliable plasma biomarker for the early detection of EAOC, as its different expression patterns can be used to distinguish endometriosis and EAOC patients from healthy controls [107]. Besides miR-21, PTEN is also a confirmed target of miR-26a and miR-214. These microRNAs are highly expressed in endometrioid and ovarian cancers, where PTEN activity is restricted, while their downregulation in endometriosis cases has tumor-suppressive effects [108]. Mir-100, a microRNA that targets mTOR, is also aberrantly expressed in EAOC. After performing deep sequencing to explore miRNA expression in 10 human OCCC cell lines, Nagaraja et al. found mir-100 to be the most under-expressed microRNA in OCCC, while its target mTOR was found to be upregulated. Interestingly, in the same study, mir-100 overexpression repressed mTOR signaling and increased the sensitivity of the OCCC cells to the mTOR inhibitor everolimus (RAD001) [109].

### 4.5. Targeting PI3K/AKT/mTOR Pathway in EAOC Treatment

According to the European Society for Medical Oncology (ESMO) and European Society of Gynaecologial Oncology (ESGO) recommendations on ovarian cancer management, the standard first-line regimen for all histologic types of ovarian cancer is chemotherapy with carboplatin and paclitaxel. Additionally, stage III–IV patients may also receive supplementary treatment with bevacizumab, a VEGF-A inhibitor, as well as Poly (ADP-ribose) polymerase (PARP) inhibitors as maintenance therapy [110]. However, since OCCCs show low sensitivity to chemotherapy [111], there is an urgent need for the development of new targeted treatment options for this ovarian cancer histotype.

In this context, given the genetic alterations in PI3K/AKT/mTOR pathway that characterize EAOCs, the potential for mTOR signaling to serve as a therapeutic target in this cancer type has been further studied. In the study of Lapke et al., the predicted actionability of mTOR in patients with EnOCs and OCCCs was 73% and 65%, respectively [93]. Mabuchi et al. examined the effects of mTOR inhibition in cisplatin-sensitive and cisplatin-resistant OCCC cell lines by treating them with everolimus (RAD001) both in vitro and in vivo. Interestingly, cisplatin-resistant cells exhibited high levels of phospho-mTOR, a fact that can be interpreted as mTOR protein activation and were more sensitive to everolimus treatment compared to cisplatin-sensitive cells, as it was observed in vitro and in vivo. The above findings opened the perspective of using everolimus as a second-line treatment for disease recurrence after cisplatin treatment [112]. Oishi et al. treated OCCC and ovarian serous adenocarcinoma (OSAC) cell lines with the dual PI3K-mTOR inhibitor NVP-BEZ235 and the selective mTOR inhibitor temsirolimus. They noted a lower IC50 value of NVP-BEZ235 in OCCC cells in comparison to OSAC cells, while the IC50 value of NVP-BEZ235 was lower than the IC50 value of temsirolimus in the OCCC cells. They also demonstrated that NVP-BEZ235 treatment induced apoptosis of OCCC cells in a concentration-dependent manner, while it reduced tumor growth in mice bearing OCCC [113]. Additionally, in the aforementioned mouse model experiment of Chandler et al., administration of the pan-PI3K inhibitor BKM120 in OCCC mice resulted in restraint of tumor cell growth and extension of animal survival, a finding that strengthened the rationale for using PI3K inhibitors in OCCC treatment [75]. Caumanns et al. demonstrated the cooperative effects of AZD8055, GDC0941, and selumetinib, which are drugs with inhibitory action against mTORC1/2, PI3K, and MEK1/2, respectively, when co-administered at a low-dose in OCCCs both in vitro and in vivo, suggesting this triple drug combination as an effective treatment strategy for this tumor type [114]. In the study of Papp et al., GNE-493-mediated PI3K pathway inhibition was more effective in ovarian cancer cell lines carrying mutations in PPP2R1A [115]. PPP2R1A is a subunit of protein phosphatase 2A (PP2A) with known tumor suppressor activity that regulates PI3K signaling via the inhibition of AKT [116]. Consistent with the findings of this study, PPP2R1A is frequently mutated in OCCCs [117], thus offering the therapeutic advantage of targeting the PI3K/AKT/mTOR pathway in this cancer type. Furthermore, Samartzis et al. observed that ARID1A loss in OCCC cells was linked to high pAKT-Ser473 levels and was associated with responsiveness to treatment with the AKT-inhibitor MK-2206, highlighting again the synergistic interconnection between ARID1A deficiency and PI3K/AKT pathway inhibition [118]. Similarly, Chien et al. identified higher sensitivity of ARID1A-deficient OCCC cell lines to the dual mTOR/PI3K inhibitor GDC0941 and the mTOR inhibitor PP242 [119]. On the other hand, data concerning other malignancies have shown a possible association between loss of ARID1A expression and resistance to PI3K/AKT/mTOR inhibitors. In fact, ARID1A loss induces annexin A1 (ANXA1) expression, which activates AKT, thereby probably promoting resistance to PI3K/AKT pathway inhibitors. Inhibition of AKT via MK2206 has been shown to reinstate the sensitivity of breast cancer cells with ARID1A deficiency to both trastuzumab and the mTOR inhibitor AZD8055 [120]. Finally, in a phase 2 clinical trial including ninety advanced-stage OCCC patients, temsirolimus was evaluated in combination with standard chemotherapy and as consolidation therapy. In that case, even though the regimen was well tolerated by the patients, no statistically significant improvement in progression-free survival was noted [121].

## 5. Conclusions

Activation of the PI3K/AKT/mTOR signaling cascade is prominent throughout the transformation process from normal endometrium to endometriosis to EAOC, as evidenced by the altered expression of key pathway components both in eutopic and ectopic endometrium. Based on this knowledge, molecules involved in mTOR signaling could be explored as future biomarkers for the prediction and prevention of endometriosis and ovarian cancer development, while further assessment of the therapeutic potential of mTOR pathway inhibitors in both endometriosis and EAOC would be necessary.

## Figures and Tables

**Table 1:** Frequency of PI3K/AKT/mTOR pathway—associated gene alterations in ovarian clear cell (OCCC) and endometrioid (EnOC) carcinomas.

Authors [Ref]	HISTOLOGICAL Type	No of Samples	ARID1A	PIK3CA	PTEN	ARID1A-PIK3CA Co-Mutations
Wang et al., 2017 [91]	OCCC	35	54% (19/35)	54% (19/35)	6% (2/35)	40% (14/35)
Itamochi et al., 2017 [85]	OCCC	55	42% (23/55)	35% (19/55)	2% (1/55)	25% (14/55)
Murakami et al., 2017 [55]	OCCC	39	62% (24/39)	51% (20/39)	5% (2/39)	NA
Shibuya et al., 2018 [82]	OCCC	48	67% (32/48)	50% (24/48)	2% (1/48)	46% (22/48)
Kim et al., 2018 [92]	OCCC	15	4% (6/15)	40% (6/15)	13% (2/15)	20% (3/15)
Yang et al., 2020 [90]	OCCC	42	64% (27/42)	29% (12/42)	7% (3/42)	26%(11/42)
Lapke et al., 2021 [93]	OCCC	23	39% (9/23)	43% (10/23)	0% (0/23)	22% (5/23)
Oliveira et al., 2021 [94]	OCCC	55	49% (27/55)	42% (23/55)	NA	36% (13/36)
Bolton et al., 2022 [95]	OCCC	421	49% (205/421)	45% (188/421)	NA	≤40% (≤167/421) *
Wang et al., 2017 [91]	EnOC	29	41% (12/29)	52% (15/29)	41% (12/29)	3% (1/36)
Cybulska et al., 2019 [59]	EnOC	36	19% (7/36)	39% (14/36)	33% (12/36)	3% (1/36)
Pierson et al., 2020 [61]	EnOC	26	19% (5/26)	27% (7/26)	46% (12/26)	12% (3/26)
Hollis et al., 2020 [60]	EnOC	112	36% (40/112)	43% (48/112)	29% (32/112)	21% (23/112)
Lapke et al., 2021 [93]	EnOC	22	32% (7/22)	32% (7/22)	27% (6/22)	23% (5/22)
Su et al., 2019 [88]	OCCC and EnOC	16	56% (9/16)	50% (8/16)	NA	44%(7/16)
Total of frequencies		974	47% (462/974)	43% (420/974)	18% (85/482)	24% (122/514)

ARID1A, AT-rich interaction domain 1A; PTEN, Phosphatase and tensin homolog; PIK3CA, Phosphatidylinositol-4,5-bisphosphate 3-kinase catalytic subunit alpha. * These data were not included in the calculation of the total rate.

## Data Availability

Data sharing is not applicable to this article.
